# Friction Compensation Control of Electromechanical Actuator Based on Neural Network Adaptive Sliding Mode

**DOI:** 10.3390/s21041508

**Published:** 2021-02-22

**Authors:** Wei Ruan, Quanlin Dong, Xiaoyue Zhang, Zhibing Li

**Affiliations:** School of Instrumentation and Optoelectronic Engineering, Beihang University, Beijing 100191, China; ruanwei@buaa.edu.cn (W.R.); dongquanlin@buaa.edu.cn (Q.D.); lizhibing@buaa.edu.cn (Z.L.)

**Keywords:** electromechanical actuator system, adaptive sliding mode controller, radial basis function neural network controller, friction compensation

## Abstract

In this paper, a radial basis neural network adaptive sliding mode controller (RBF−NN ASMC) for nonlinear electromechanical actuator systems is proposed. The radial basis function neural network (RBF−NN) control algorithm is used to compensate for the friction disturbance torque in the electromechanical actuator system. An adaptive law was used to adjust the weights of the neural network to achieve real−time compensation of friction. The sliding mode controller is designed to suppress the model uncertainty and external disturbance effects of the electromechanical actuator system. The stability of the RBF−NN ASMC is analyzed by Lyapunov’s stability theory, and the effectiveness of this method is verified by simulation. The results show that the control strategy not only has a better compensation effect on friction but also has better anti−interference ability, which makes the electromechanical actuator system have better steady−state and dynamic performance.

## 1. Introduction

The trajectory correction projectile is based on the original projectile and replaced with a guidance part, so that it has the ability to accurately strike. As the actuator of the trajectory correction projectile, the main function of the electromechanical actuator (EMA) system is to realize the tracking control of the commanded angular position. During the flight of the projectile, it needs to continuously adjust its attitude according to the target position. Therefore, the tracking speed and tracking accuracy of the EMA system will have a vital influence on the mobility and accuracy of the trajectory correction projectile. Compared with hydraulic actuators and pneumatic actuators, electromechanical actuators are widely used because of their simple structure, convenient control, and low cost [1].

The EMA system is mainly composed of a controller, a driver, a Brushless DC (BLDC) motor, a ball screw reducer, a speed sensor, and a position sensor [2]. Therefore, the system will inevitably be adversely affected by friction during transmission. Especially in the case of low speed, it will cause a dead zone, crawling, and tracking error to adversely affect the control performance of the system [3]. In addition, due to the uncertainty of the model and the existence of external disturbances, it brings serious difficulties and challenges to the design of high−performance EMA system controllers.

In order to overcome the influence of friction disturbance on the performance of the control system, scholars have designed many compensation techniques to improve the performance of the nonlinear servo control system. Generally, there are two main methods of friction compensation [4]. The first is to design a compensation controller based on the friction model. Another method is to treat the friction torque disturbance as an external disturbance and use an intelligent control strategy to suppress it. Common friction models are mainly divided into static friction models and dynamic friction models [5]. Among them, static friction models mainly include the Coulomb model [6], Stribeck model [7], Karnopp model [8], and Armstrong model [3]. The static friction model has a simple structure and the parameters in the model are easy to identify, but it cannot accurately describe the friction phenomenon, so the friction compensation based on the static model has limited performance improvement of the system. Therefore, various scholars have also carried out research on dynamic friction models, mainly including the Dahl model [9], Elasto−plastic model [10], LuGre model [11], and Leuven model [12]. These dynamic models can describe the friction phenomenon relatively accurately, but the parameters of the dynamic model are complex and difficult to identify. Based on the friction model compensation, scholars have also conducted extensive research. Xu, L., and B. Yao designed an adaptive robust controller based on the LuGre friction model to improve the effect of dynamic friction on system performance [13]. Tong, H.L built a system’s adaptive compensator based on the LuGre friction [14]. Based on the LuGre friction model, Huang used neural network approximation to compensate for friction, so as to obtain good tracking performance [15]. Feng proposed a friction feedforward compensation method based on the improved Stribeck model, which effectively eliminated the low−speed creep and amplitude flattening phenomenon in the dispensing servo system, and improved the control performance of the system [16]. Based on the Stribeck friction model, X.Y. studied and compared the precision of position and velocity controlled by PID control and BP neural network when the seeker platform was working at the low speed [17].

In the method of adopting an intelligent control strategy, people have studied many advanced nonlinear control strategies. Common methods include sliding mode control, adaptive integral backstepping sliding mode control, and dimensionality reduction observation. Wang designed the controller of the robot by adopting the terminal sliding mode control strategy of a neural network to suppress the influence of model uncertainty and external interference [18]. An adaptive neural network sliding mode scheme was used in the robot trajectory tracking system [19]. An adaptive actor−critic controller was designed for some nonlinear systems with unknown input disturbances [20]. Seong Ik Han designed a recurrent fuzzy neural network and reconstructed error compensator as well as a robust friction state observer to achieve high−precision positioning performance of mechanical systems [21]. In the above−mentioned various intelligent control strategies, there will always be errors in the observation and approximation of friction disturbances, and there will be interferences from other nonlinear factors in the system. Therefore, in order to obtain better control performance, a new control strategy needs to be introduced on this basis. While compensating for friction, the new control strategy can eliminate the effects of compensation errors and other nonlinear disturbances.

In addition, with the development of control technology and the continuous improvement of control requirements, some new control technologies have also been proposed. In [22], a long−distance teleoperation control system, signal delay and uncertainty are inevitable problems. This paper investigated the feasibility of a more straightforward, classical control solution based on Kessler’s Extended Symmetrical Method, formulated in a cascade control approach to tackle the problems caused by latency and uncertainties in a modeled telesurgical robot system. In order to get a better vibration isolation capability than other methods, in [23] a combination of skyhook and groundhook control−based magneto rheological lookup table technique called hybrid control for a quarter car was developed. In [24], a data−driven model−free sliding mode learning control (MFSMLC) for a class of discrete−time nonlinear systems was proposed. This method does not require a specific mathematical model, and in addition the chattering is reduced because there is no non−smooth term in the controller. It can be seen from the above literature that there are different control strategies for different systems. Therefore, in view of the friction and other nonlinear disturbances in the EMA system, this paper focuses on finding an effective control strategy to improve the performance of the system.

Because the neural network has good approximation characteristics, it is a relatively advanced method for tracking control of nonlinear dynamic systems. For the approximation error of the neural network, the sliding mode control strategy is often used to compensate. Lewis designed a neural network control method for the robot system [25]. Jui−Hong presented a neural network−based adaptive control strategy for speed or position tracking of a DC motor with unknown system nonlinearities [26]. Nasser Sadati proposed a robot adaptive multimodel sliding mode controller; a radial basis function neural network (RBF−NN) was used to approximate the discontinuous part of the control signal [27]. Faa−Jeng Lin designed a robust dynamic sliding mode controller for the magnetic levitation system and used a linear neural network estimator to estimate an unknown nonlinear function with lumped uncertainty online [28]. For nonholonomic wheeled mobile robot systems, Bong Seok Park proposed an adaptive neural sliding mode control method. Autoregressive wavelet neural networks are used to approximate arbitrary model uncertainties and external disturbances in mobile robot dynamics [19].

In [29], the advantages and disadvantages of eight common friction models are listed. In engineering applications, the LuGre friction model or the Stribeck friction model is often used to compensate the friction in the system. However, due to the complex internal parameters of the LuGre friction model, it is difficult to identify the parameters in the model in practical applications. As for the electric steering gear system, since its interior is reciprocating, the rotation speed of the system is slow, so the Stribeck friction model can also accurately reflect the friction disturbance torque in the system. In addition, the neural network used in this paper can be adjusted online, and the sliding mode controller can compensate for the approximation error of the model. For this reason, this paper proposes an RBF−NN adaptive sliding mode controller (ASMC). Aiming at the friction disturbance, based on the Stribeck friction model, the neural network adaptive method is adopted to approximate the friction torque, so as to realize the compensation of the friction torque. Aiming at the approximation error and other nonlinear disturbances, a sliding mode control strategy is adopted to improve the control performance of the system. Through the adaptive learning method, the closed−loop system can be guaranteed to be globally stable.

The rest of this article is organized as follows: The second part of this article establishes the dynamic model of the EMA system based on the system composition and principles, and analyzes the characteristics of the Stribeck friction model. The third part of this paper designs a sliding mode controller based on the system model and designs a neural network adaptive controller to approximate the friction torque, and finally proves the stability of the controller. The fourth part of this article is to build a simulation model in MATLAB/Simulink, design a simulation algorithm to simulate the controller, and finally compare the simulation results with a sliding mode controller to analyze the performance of the system.

## 2. Materials and Methods

### 2.1. System Composition and Working Principle

**Figure 1:**
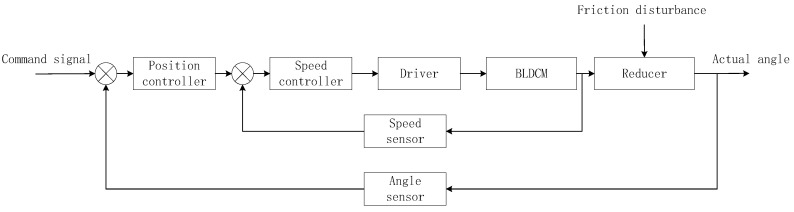
Structure diagram of the electromechanical actuator (EMA) system.

The EMA system studied in this article is composed of a controller, a driver, a BLDC motor, a ball screw reducer, a speed sensor, and an angle sensor. Figure 1 shows the composition of the EMA system. The working principle is: The controller generates a control voltage according to the deviation between the command signal and the actual signal. The driver amplifies the controller to drive the BLDC motor to rotate. The BLDC motor transmits torque through the ball screw reducer to drive the rudder wing to the specified position [30].

In the EMA system, due to the existence of transmission mechanisms such as reducers, there will inevitably be the influence of frictional disturbance torque during the transmission process. Generally, friction disturbance mainly acts on the transmission mechanism. In this paper, in order to analyze and design a suitable controller, the friction disturbance torque is equivalent to the output shaft of the BLDC motor.

### 2.2. EMA System Modeling

#### 2.2.1. BLDC Motor Modeling

The motor is an electrical component of the entire system, and its performance directly affects the performance of the system. What this text chooses is BLDC electrical machinery—it has the characteristic of a small moment of inertia, and high speed meets the requirement of the fast response of the system [31]. Under ideal conditions, the armature circuit balance equation of the motor is

(1) $$u(t)=E+Ri_{d}(t)+L\frac{di_{d}(t)}{dt},$$

where, *u*(*t*) is the armature voltage of the motor, *i_d_* is the armature current, *R* the motor armature resistance, *L* is the inductance of the motor armature, and *E* is a counter electromotive force of the motor armature.

The induced electromotive force of the motor is proportional to the motor speed, which is

(2) $$E=C_{e}\omega (t),$$

where, *C_e_* is the back electromotive force coefficient, *ω*(*t*) is the angular velocity of the motor.

According to Newton’s second law, the dynamic equation of the motor is

(3) $$T_{e}-T_{L}=J\frac{d\omega }{dt},$$

where, $T_{e}=C_{m}i_{d}(t)$ is the electromagnetic torque, *T_L_* is the load torque, *C_m_* is the electromagnetic torque coefficient, and *J* is the moment of inertia.

When the external load is 0, the above formulas are combined and organized, and the pull transformation is performed to obtain the open−loop transfer function of the motor as

(4) $$\frac{\omega (s)}{U(s)}=\frac{1/C_{e}}{\tau_{l}\tau_{m}s^{2}+\tau_{m}s+1},$$

where, $\tau_{m}=\frac{RJ}{C_{e}C_{m}}$ is the Electromechanical time constant and $\tau_{l}=\frac{L}{R}$ is the Electromagnetic time constant.

#### 2.2.2. Drive and Reducer Modeling

The output voltage of the controller cannot directly drive the BLDC motor, so the driver is required to amplify the signal of the controller. The drive circuit is composed of field−effect tubes, so for the drive, its transfer function can be expressed as

(5) $$G_{p}(s)=\frac{K_{p}}{Ts+1},$$

where, *K_P_* is the magnification of the driver, and *T* is the delay time constant of the field−effect tube. Under normal circumstances, the delay time of the FET is much smaller than the delay time of the controller, so the mathematical model of the driver can be further simplified as

(6) $$G_{p}(s)=K_{p},$$

The motor and the rudder wing are connected by a ball screw reducer, so the approximate relationship between the rotation angle of the rudder blade and the motor angle is

(7) $$\frac{d\theta }{dt}=\frac{\omega }{i},$$

where, *θ* is the deflection angle of the rudder wing and *i* is the reduction ratio of the EMA system reducer.

Therefore, the transfer function of the reducer part can be expressed as

(8) $$\theta (s)=\frac{\omega (s)}{is},$$

#### 2.2.3. Stribeck Friction Model

Because the frictional disturbance torque in the EMA system has a high degree of nonlinearity and complexity, it has a greater impact on the control performance of the system, causing the system to crawl at low speeds, and there will be larger errors in the steady−state. The Strobeck friction model is the most common friction model in engineering, which can fully and accurately reflect the friction phenomenon in the EMA system. The expression of the disturbance torque is [32]

(9) $$T_{f}(\omega )=T_{l}\cdot sign(\omega )+(T_{s}-T_{l})e^{-{\left|\omega /\omega_{s}\right|}^{2}}+b\omega ,$$

where, *T_f_* is the friction torque of the system, *T_l_* is the Coulomb friction torque, *T_s_* is the maximum static friction torque, ω is the angular velocity of the motor output shaft, *ω_s_* is the Stribeck speed, and *b* is the viscous friction coefficient.

Generally, friction models are divided into continuous and discontinuous models. The main difference is whether it is continuous at zero speed [33]. The Stribeck friction model used in this article is a discontinuous model, so it has the problem of speed zero crossing detection. In the Stribeck friction model, a symbolic function is used. Since the generalized zero velocity is difficult to measure, the saturation function is used instead of the sign function to solve the problem of velocity zero crossing detection.2.2.3 EMA system model.

According to the above analysis of each part of the EMA system, according to the working principle of the EMA system, when the external load is 0, the open−loop transfer function of the EMA system can be obtained as

(10) $$\begin{array}{ll} G & =G_{p}\cdot \frac{\omega (s)}{U(s)}\cdot \frac{\theta (s)}{\omega (s)}\\ & =\frac{K_{p}/(C_{e}\cdot i)}{\tau_{l}\tau_{m}s^{3}+\tau_{m}s^{2}+s} \end{array},$$

**Figure 2:**
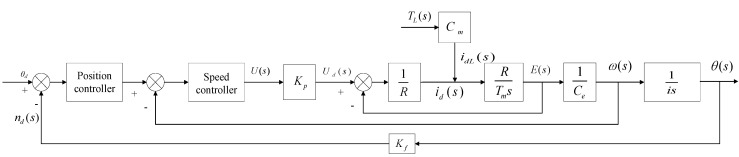
Block diagram of the open-loop transfer function of the EMA system.

According to the above analysis and the open−loop transfer function of the system, the overall control block diagram of the system can be obtained, as shown in Figure 2.

In Figure 2, *T_L_* is the sum of frictional disturbance torque equivalent and other load disturbances to the motor output shaft.

### 2.3. Controller Design

#### 2.3.1. Controller Structure

Take $x=[x1,x2]=[\theta ,\dot{\theta }]$, for the EMA system, $\tau_{l}\ll \tau_{m}$, the EMA system can be simplified into a second−order system. Consider the friction disturbance, the system model can be expressed by Equation (11).

(11) $$\ddot{\theta }=-\frac{1}{\tau_{m}}\dot{\theta }+\frac{K_{p}}{\tau_{m}C_{e}i}(u-T_{f}(x))+d(t)=f(x)+p(u-T_{f}(x))+d(t),$$

where, *d*(*t*) is the external disturbance, and, *d*(*t*) < D, D is a constant, and $T_{f}(x)$ is the friction disturbance in the EMA system. And, $f(x)=-\frac{1}{\tau_{m}}$, $p=\frac{K_{p}}{\tau_{m}C_{e}i}$.

**Figure 3:**
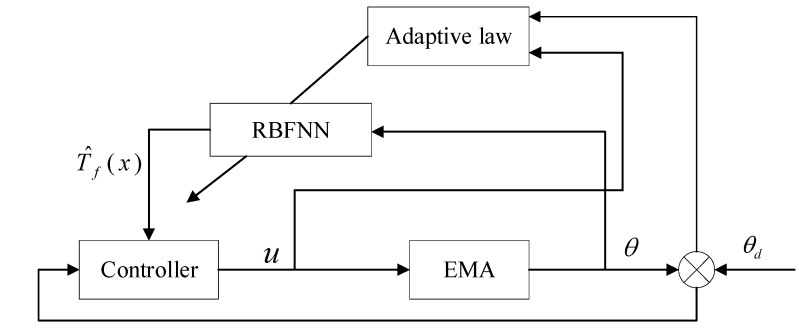
RBF-NN ASMC block diagram.

The structure of the RBF−NN ASMC designed in this paper is shown in Figure 3.

Because sliding mode control has the characteristics of insensitivity to parameters and strong antidisturbance, for the EMA system, sliding mode control is used to realize the tracking control of the position signal to overcome the uncertainty of the system model and the influence of external disturbances. For the compensation of friction disturbance torque, on the basis of the Stribeck friction model, the RBF−NN method is used to approximate the friction torque. The adaptive law is used to adjust the weights of the network to achieve the global stability of the system.

#### 2.3.2. Controller Design

In this paper, the Gaussian function is used as the hidden layer of the RBF−NN, and the output of the neural network can be expressed as

(12) $$Y=\sum_{j=1}^{m}w_{j}h_{j}+b=W^{T}H+b,$$

The expression of Gaussian function is

(13) $$h_{j}=\exp\left(-\frac{{\left\|X-c_{j}\right\|}^{2}}{2\sigma_{j}^{2}}\right),$$

In the formula, $c_{j}$ is the center of the Gaussian function; $\sigma_{j}$ represents the width of the Gaussian function. $w_{j}$ is the weight from the hidden layer to the output layer; b is the bias of the output neuron.

In this paper, a neural network is used to approximate the frictional disturbance torque. Therefore, the frictional torque can be expressed by the output of the neural network

(14) $$T_{f}(x)=W^{*T}h(x)+\varepsilon ,$$

where, $W^{*}$ is the ideal network weight of the neural network, ε is the error of the ideal neural network approximation, and $\left|\varepsilon \right|\leq \varepsilon_{\max}$.

Take the estimated value of $W^{*}$ as $\hat{W}$ then the estimated value of $T_{f}(x)$ can be expressed as

(15) $${\hat{T}}_{f}(x)={\hat{W}}^{T}h(x),$$

Then the estimated error of the network weight is $\tilde{W}=W^{*}-\hat{W}$.

Take $x_{1}=\theta$, let the ideal angular position signal be $\theta_{d}$, then the angular position error is $e=\theta_{d}-\theta$, and the sliding mode function of the EMA system is

(16) $$s=\dot{e}+ce,$$

Derivation of sliding mode function of the EMA system

(17) $$\begin{array}{ll} \dot{s} & =\ddot{e}+c\dot{e}={\ddot{\theta }}_{d}-\ddot{\theta }+c\dot{e}\\ & ={\ddot{\theta }}_{d}-f(x)-p(u-T_{f}(x))-d(t)+c\dot{e} \end{array}$$

Equation (18) is the designed sliding mode control law

(18) $$u=\frac{1}{p}(f(x)-{\ddot{\theta }}_{d}+c\dot{e}+\eta \mathrm{sgn}(s))+{\hat{T}}_{f}(x),$$

where, $\eta \geq D+p\varepsilon_{\max}$.

Based on the above equations, Equation (17) can be further simplified

(19) $$\begin{array}{ll} \dot{s} & ={\ddot{\theta }}_{d}-f(x)-p(u-T_{f}(x))-d(t)+c\dot{e}\\ & ={\ddot{\theta }}_{d}-f(x)-p((\frac{1}{p}(f(x)-{\ddot{\theta }}_{d}+c\dot{e}+\eta \mathrm{sgn}(s))+{\hat{T}}_{f}(x))-T_{f}(x))-d(t)+c\dot{e}\\ & =-\eta \mathrm{sgn}(s)+p({\hat{T}}_{f}(x)-T_{f}(x))+d(t)\\ & =-\eta \mathrm{sgn}(s)+p{\tilde{T}}_{f}(x)+d(t)\\ & =-\eta \mathrm{sgn}(s)+p({\tilde{W}}^{T}h-\varepsilon )+d(t) \end{array}$$

#### 2.3.3. Proof of Controller Stability

Define the Lyapunov function as

(20) $$V=\frac{1}{2}s^{2}+\frac{1}{2\gamma }{\tilde{W}}^{T}\tilde{W},$$

where, γ is the adaptive gain and γ>0.

Take the time−derivative of and simplify Equation (20)

(21) $$\begin{array}{ll} \dot{V} & =s\dot{s}+\gamma {\tilde{W}}^{T}\dot{\hat{W}}\\ & =s(-\eta \mathrm{sgn}(s)+p({\tilde{W}}^{T}h-\varepsilon )+d(t))+\frac{1}{\gamma }{\tilde{W}}^{T}\dot{\tilde{W}}\\ & =-\eta \left|s\right|+sd(t)-sp\varepsilon +{\tilde{W}}^{T}(sph+\gamma \dot{\hat{W}}) \end{array}$$

Take the adaptive law as

(22) $$\dot{\hat{W}}=-\frac{1}{\gamma }sph(x),$$

Substitute Equation (22) into Equation (21) and simplify

(23) $$\dot{V}=-\eta \left|s\right|+s(d(t)-p\varepsilon )\leq 0,$$

Take $\dot{V}\equiv 0$, according to LaSalle invariant set theorem, when t→∞, s→0. Therefore, the controller designed in this article is stable.

## 3. Simulation Results and Analysis

### 3.1. Parameter Settings

The parameters of the EMA system are shown in Table 1.

The parameters in the Stribeck friction model are set as: $T_{f}=15$, $T_{s}=20$, b=0.4, $\omega_{s}=0.05$. Therefore, the frictional disturbance torque of the EMA system is

(24) $$T_{f}(\omega )=15\cdot \mathrm{sgn}(\omega )+5\cdot e^{-{\left(\omega /0.05\right)}^{2}}+0.4\omega ,$$

In order to verify the performance of the RBF−NN ASMC, this paper also designs a sliding mode controller based on the exponential reaching law. To this end, this article builds a model of the EMA system in MATLAB and Simulink, and uses the S function to implement two control algorithms.

The ideal angle signal of the system is taken as $\theta_{d}=\sin(t)$, and take the external disturbance as d(t)=sin(t). The initial state is taken as [0.2,0]. For the RBF−NN, the structure of 1−5−1 is adopted, the input of the network is *x*_2_, and the center vector of the Gaussian function is designed as $c_{i}=[-1,-0.5,0,0.5,1]$ and the width is $b_{j}=1$. The initial weight of the network is 0, and the adaptive gain is γ=0.1. For the sliding mode controller based on the exponential reaching law, the friction disturbance is treated as an external disturbance. The EMA system is simulated by adjusting the parameters of each controller.

### 3.2. Simulation Results

In order to verify the pros and cons of the RBF−NN ASMC performance, this article starts from three situations to simulate and analyze the system. The first is to simulate the Sliding mode controller(SMC) based on the reaching law without considering the friction disturbance to verify the robustness of the sliding mode controller to model uncertainty and load disturbance. Then, considering the impact of friction disturbance on the EMA system, the SMC based on the reaching law and the RBF−NN ASMC is simulated respectively.

#### 3.2.1. Simulation of SMC without Friction Disturbance

**Figure 4:**
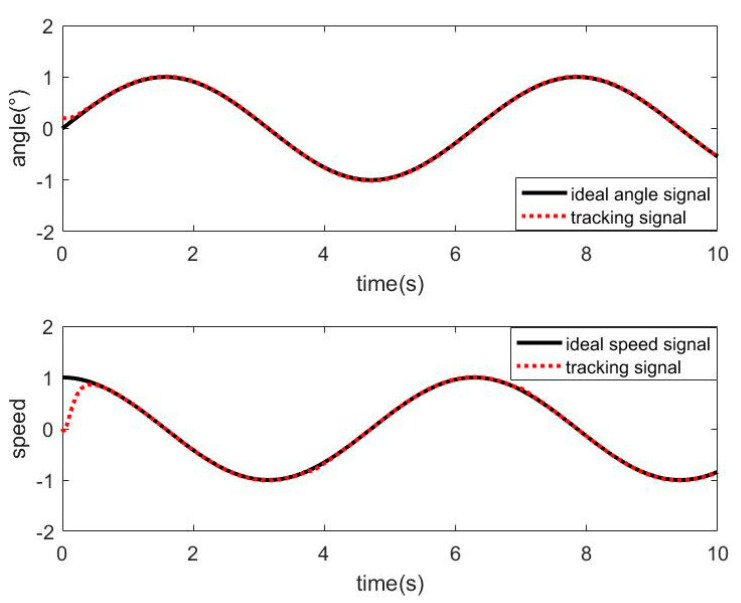
Tracking curve of SMC without friction disturbance.

**Figure 5:**
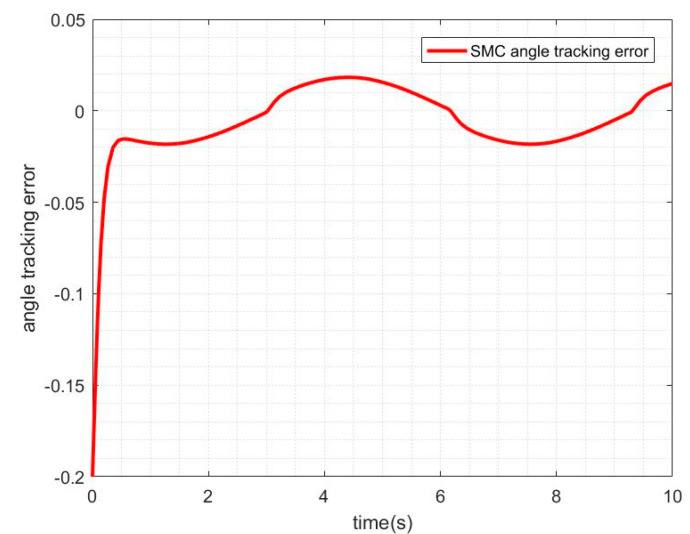
SMC angle-tracking error without friction disturbance.

When the friction disturbance is zero, the sliding mode controller with exponential reaching law is used to control the system, and its angle and velocity tracking trajectory are shown in Figure 4. The angle tracking error curve is shown in Figure 5.

It can be seen from the above two figures that the sliding mode controller has good performance for nonlinear systems, has a fast response to command signals and small tracking errors, but it can be seen from the error−tracking curve that there is a comparison in the control process.

#### 3.2.2. Simulation of SMC with Friction Disturbance

**Figure 6:**
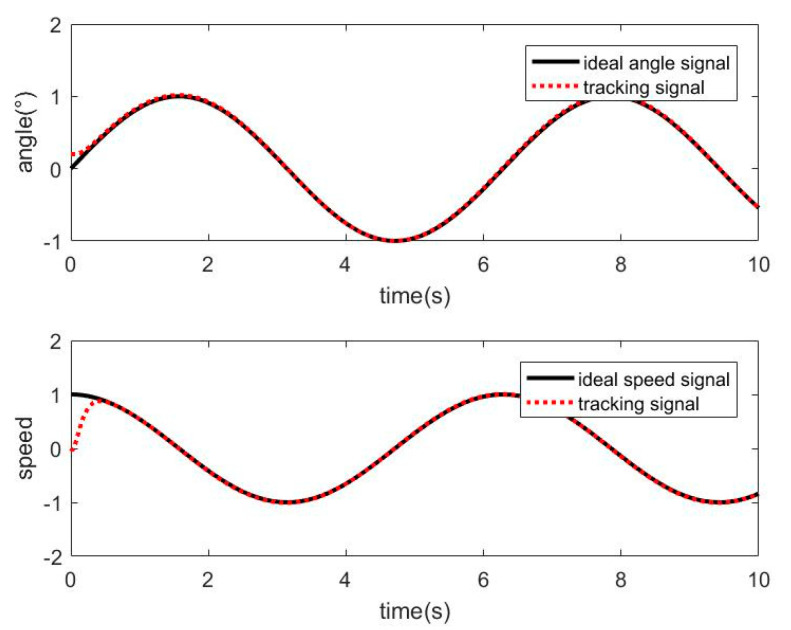
Tracking curve of SMC with friction disturbance.

**Figure 7:**
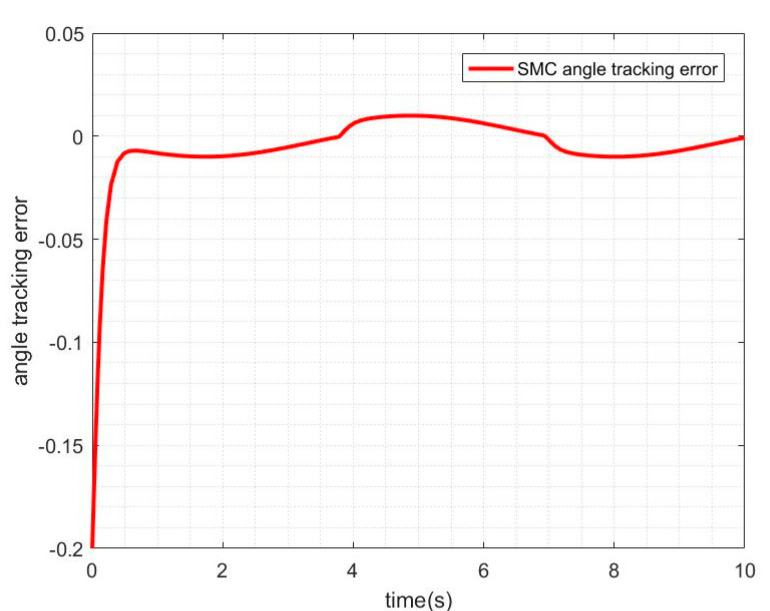
SMC angle tracking error with friction disturbance.

The friction disturbance torque is added to the control system, and the SMC method is used for simulation. The tracking curve of angle and speed is shown in Figure 6, and the angle tracking error is shown in Figure 7.

**Figure 8:**
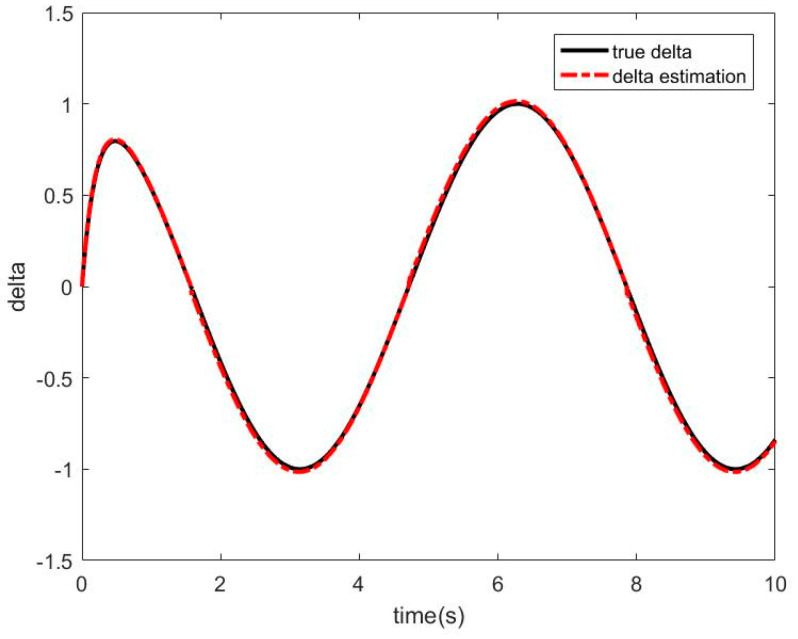
Approximation curve of RBF-NN to frictional disturbance torque.

It can be seen from Figure 7 and Figure 8 that when there is friction disturbance, the control system still has a good effect on tracking position and speed, but when compared with no friction disturbance, the performance of sliding mode control has been reduced; especially in the control process, the tracking error is larger.

#### 3.2.3. Simulation of RBF−NN ASMC with Friction Disturbance

**Figure 9:**
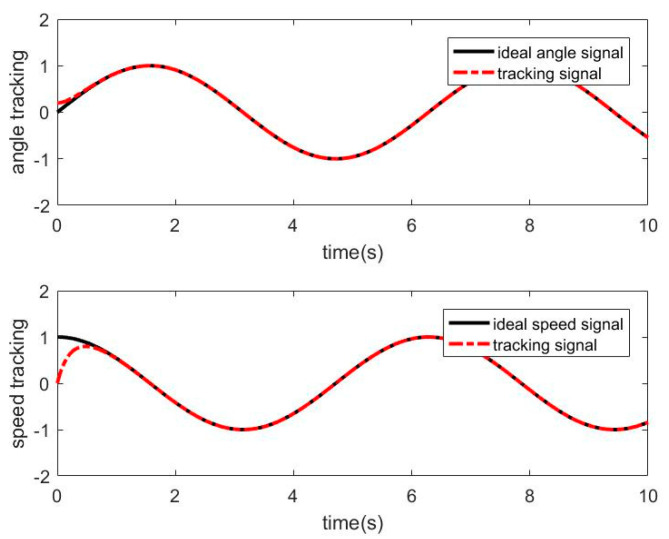
Tracking curve of RBF-NN ASMC with friction disturbance.

**Figure 10:**
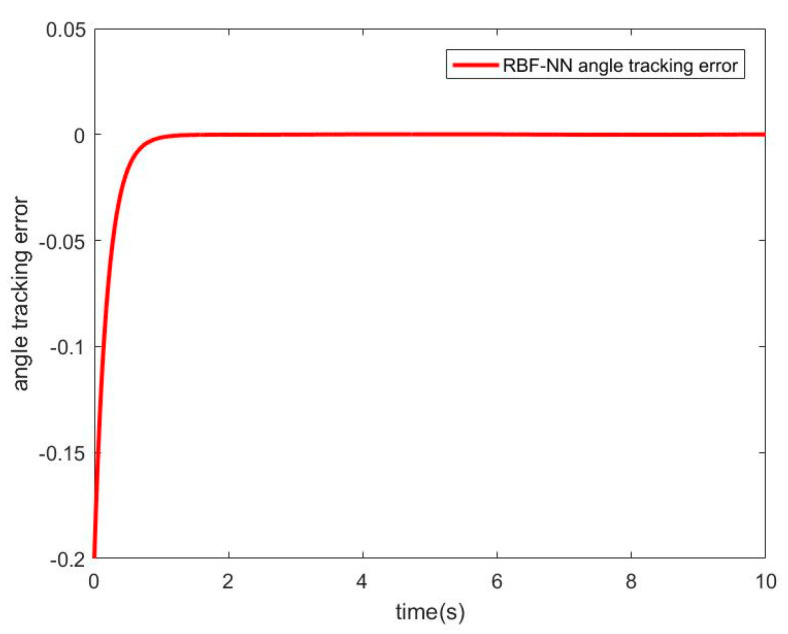
RBF--NN ASMC angle tracking error with friction disturbance.

When there is friction disturbance, the RBF−NN is used to approximate the friction disturbance torque, and the SMC is used for compensation control. The simulation results obtained are as follows. Figure 8 shows the approximation curve of the RBF−NN to frictional disturbance torque. Figure 9 shows the position and speed tracking curve of the RBFNN−ASMC. Figure 10 shows the angular position tracking error curve.

It can be seen from Figure 8 that RBF−NN can have a better effect on the approximation of the friction disturbance torque. It can be seen from Figure 9 and Figure 10 that the RBF−NN ASMC also has a good position and speed tracking effects, and from the error curve, it can be seen that its tracking error is smaller than that of the SMC. And it also has a certain inhibitory effect on the chattering problem of the SMC.

### 3.3. Simulation Result Analysis

From the simulation results, it can be seen that when the friction disturbance of the system is not considered, the SMC can achieve a better control effect on the nonlinear system, but when the friction disturbance is introduced into the system, the quality of the SMC decreases. When RBF−NN is used to approximate the frictional disturbance torque and the SMC is used to compensate and control the whole, not only can it achieve a good approximation effect on the friction disturbance, but also the overall control performance of the system has improved. In addition, because the neural network compensates for the friction in the system, for the sliding mode controller, the chattering phenomenon can also be suppressed to a certain extent. The chattering problem inherent in the SMC is solved. Therefore, it can be seen from the simulation results that the RBF−NN ASMC proposed in this paper can not only compensate for the friction disturbance but also have a better control effect on other nonlinear factors in the system.

In the related literature investigated in this article, people have proposed various methods to solve the friction and other nonlinear problems in the EMA system, but due to the inconsistency of system parameters and simulation methods, the simulation results have little reference significance. For example, in [34], an adaptive fuzzy sliding mode control strategy to suppress the parameter perturbation and external interference of the EMA system is used, but from the simulation results, its tracking performance for sinusoidal signals is not as good as this article’s designed controller. Compared with other adaptive control systems, the advantage of this paper is that the neural network is used to compensate the friction disturbance in the system, and the adaptive method enables the neural network to have the ability of online learning. Finally, the compensation error and other disturbances are compensated by sliding mode control. Therefore, the RBF−NN ASMC proposed in this paper can improve the performance of the EMA system to a certain extent and provide an idea for solving the friction disturbance and other nonlinear disturbances in the system.

## 4. Conclusions

Aiming at the nonlinearity in the EMA system and the influence of friction disturbance, this paper proposes a control strategy combining an RBF neural network and adaptive sliding mode controller. First, the friction phenomenon in the EMA system based on the Stribeck friction model is analyzed. Next, an RBF neural network method is used to approximate the friction disturbance torque. Then, this paper designs an adaptive law to adjust the weight of the neural network so that the friction model can be adjusted according to the actual situation. On this basis, the SMC is designed to compensate for the approximation error of the RBF−−NN to frictional disturbance torque. In addition, considering the uncertainty and nonlinearity of other parts of the system, the SMC also has a better control performance. The simulation results show that the developed RBF−NN SMC can compensate for friction well, and also has a good control performance on other nonlinear and uncertain factors in the system. Also, the inherent chattering problem of the SMC controller is also suppressed due to the existence of the RBF−NN controller. Therefore, the proposed RBF−NN SMC has better compensation and suppression for friction and other disturbances in the EMA system.

## Figures and Tables

**Table 1:** Parameters of the EMA system.

Parameter	Value
Rated voltage of BLDC MOTOR	24 V
Rated speed of BLDC MOTOR	22,000 rpm
Torque constant of BLDC MOTOR	3.6 mNm/A
Electromechanical time constant	5 ms
Armature circuit resistance	0.9 Ω
Armature loop inductance	0.05 mH
Back electromotive force constant	2.5 Vs/rad
Reduction ratio	6
Drive magnification	60

## Data Availability

The data presented in this study are available on request from the corresponding author.
